# Physiological responses to known intake of ergot alkaloids by steers at environmental temperatures within or greater than their thermoneutral zone

**DOI:** 10.3389/fchem.2014.00096

**Published:** 2014-11-12

**Authors:** Joan H. Eisemann, Gerald B. Huntington, Megan Williamson, Michelle Hanna, Matthew Poore

**Affiliations:** Department of Animal Science, North Carolina State UniversityRaleigh, NC, USA

**Keywords:** steers, tall fescue, ergot alkaloids, environmental temperature, hemodynamics, metabolism

## Abstract

Two studies separated effects of dietary ergot alkaloids from effects of feed intake or ambient temperature on respiration rate (RR), heart rate (HR), surface temperature (ST), rectal temperature (RT), blood pressure (BP), serum hormone, and plasma metabolite concentrations in beef steers. The balanced, single reversal design for each experiment used 8 beef steers fed tall fescue seed [2.5 g/kg body weight (BW)] with (E+) or without (E−) ergot alkaloids as part of a 60:40 switchgrass hay: supplement diet. Periods were 35 days with 21 days of preliminary phase and 14 days of feeding fescue seed once daily. Measures of dependent variables were collected on d 20, 25, 29, and 35 of each period at 0730 (before feeding), 1230 and 1530. In Experiment 1 steers weighed 286 kg, gained 0.61 kg BW/day, E+ supplied 2.72 mg ergot alkaloids including 1.60 mg ergovaline per steer daily, and mean minimum and maximum daily ambient temperatures were 23.6 and 32.3°C. In Experiment 2 steers weighed 348 kg, gained 1.03 kg BW/day, E+ supplied 3.06 mg ergot alkaloids including 2.00 mg ergovaline daily, and mean minimum and maximum daily ambient temperatures were 11.9 and 17.4°C. Dry matter intake was not affected by fescue seed treatment (*P* < 0.20) in either experiment. In both experiments, E+ reduced HR (*P* < 0.01) and increased insulin (*P* = 0.07). Systolic BP minus diastolic BP decreased (*P* < 0.05) for E+ in both experiments, due to increased diastolic BP in Experiment 1 (*P* < 0.03) and decreased systolic BP in Experiment 2 (*P* < 0.07). In Experiment 1, above the thermoneutral zone, E+ increased (*P* < 0.05) RR, RT, and left side ST in comparison to E−, but in Experiment 2, within the thermoneutral zone, E+ and E− did not differ (*P* < 0.18). Ergot alkaloids from fescue seed affect the cardiovascular system of steers separately from effects of feed intake or environmental temperature. Ergot alkaloids interact with ambient temperatures above the steers' thermoneutral zone to exacerbate the symptoms of hyperthermic stress.

## Introduction

Consumption of toxic, endophyte-infected, tall fescue results in ingestion and absorption of ergot alkaloids produced by the endophyte, *Neotyphodium coenophialum*, which causes fescue toxicosis in grazing cattle. Ergovaline, the alkaloid produced in greatest concentration, or total ergot alkaloids have been measured in fescue to describe its potential toxicity. Concentrations of ergovaline increased from 250 to 450–500 μg/kg in leaf blades and from 500 to 800–1300 μg/kg in leaf sheaths from April to May. Seed heads contained the greatest concentration of toxins and reached concentrations as high as 5000 μg/kg in June (Rottinghaus et al., 1991). Total ergot alkaloid concentration showed the same seasonal changes as ergovaline (Hill et al., 2000).

Studies with steers or heifers consuming different sources of fescue hay (Hemken et al., 1981) or consuming alkaloids from fescue seed (Burke et al., 2001) at differing ambient temperatures in a factorial experimental design indicate a greater response to diets with endophyte-infected vs. endophyte-free fescue (increased respiration and rectal temperatures (RTs) and decreased voluntary intake) during hyperthermic heat stress compared to ambient temperatures within the animals' thermoneutral zone. Adverse responses of cattle consuming endophyte-infected tall fescue, including lower tolerance to ambient temperatures outside the animals' thermoneutral zone, decreased voluntary intake, weight gain, and milk production have been linked to hemodynamic effects of ergot alkaloids in the tall fescue (Strickland et al., 2011). The alkaloids, particularly ergovaline, ergovalanine, and ergonovine administered i.v. (Oliver et al., 1994; Browning and Leite-Browning, 1997; Browning, 2000) or fed at doses comparable to amounts of alkaloids ingested as endophyte-infected fescue seed (Rhodes et al., 1991; Aiken et al., 2007, 2009), decreased heart rate (HR), increased blood pressure (BP), and caused vasoconstriction in steers or heifers. The responses include decreased skin temperature or increased RT and increased respiration rate (RR) at ambient temperatures greater than the animals' thermoneutral zone. Usually, ingestion or administration of alkaloids decreased serum or plasma prolactin concentrations. In a thermoneutral environment, the hemodynamic responses appeared to be muted or not detectable.

Efforts to delineate potential interactions between hemodynamic effects of alkaloid and ambient temperatures above the animals' thermoneutral zone have been complicated by concomitant changes in voluntary intake when animals consume endophyte-infected tall fescue (Hemken et al., 1981; Boling et al., 1989; Aiken et al., 2007, 2009). Additionally, close human contact required to obtain physiological measures may itself alter the animals' response and contribute to variation in response to dose levels or duration of experimental protocol (Aiken et al., 2007); therefore, most reported studies describe acute responses over a period of hours or a few days. Some researchers have addressed this potential problem by adapting animals to facilities and conditions prior to experiments, e.g., Browning (2000).

The main objectives of the current experiments were to separate the pharmacological effects of endophyte alkaloids from effects of ambient temperatures above the animals' thermoneutral zone and effects attributable to changes in intake or discomfort due to close human contact.

## Materials and methods

Two experiments were conducted under the supervision and approval of the university animal care and use committee. Angus steers from the North Carolina State University Beef Education Unit university farm of known pedigree, age, and background were trained to be led by halter and accustomed to close human contact while eating a hay diet devoid of fescue. Experiment 1 was in June to August, 2011, and Experiment 2 was in October to December, 2012. Each experiment used 8 steers. In Experiment 1 mean ± *SD* steers' age was 247 ± 24 d and in Experiment 2 it was 380 ± 27 d. Steers were housed in individual stalls with a chain attached to their halter. The stalls were 115 × 178 cm, with automatic waterers and rubber mats on concrete floors. The daily protocol was removal of orts (if any), feed supplement at 0830 h, move steers outside for exercise in a common pen for about 1 h, then back to stalls for morning hay feeding by about 1000 h. At 1530 h, the second ration of supplement was fed, followed by the second ration of hay. Lights in the barn were 18 h on: 6 h off each day, with adjustment of on and off times to accommodate the season of the year. Steers were weighed weekly, feed and orts recorded daily. Steers' stall assignment was determined when they were randomly allocated to the treatment protocol.

All steers were fed sliced switchgrass hay (Table 1) at daily amounts equal to 10 g/kg BW and a supplement, each divided into AM and PM feedings. The hay was stored in rectangular bales, and was pressed through a Van Dale Bale Processor, Model S600 (J-star industries, Ft. Atkinson WI) with knives spaced 12.5 cm apart.

**Table 1:** **Organic matter (OM), crude protein (CP), neutral detergent fiber (NDF) and acid detergent fiber (ADF) concentrations, g/kg dry matter, in feedstuffs for the 2 experiments**.

**Item**	**OM**	**CP**	**NDF**	**ADF**
Soybean hulls	949	164	533	385
Endophyte-infected seed	937	152	440	248
Endophyte-free seed	933	132	389	231
**SUPPLEMENT**				
Experiment 1	946	191	191	125
Experiment 2	945	206	191	125
**SWITCHGRASS HAY**				
Experiment 1	959	56	691	393
Experiment 2	960	74	707	390

Each experiment had two, 35-d periods with 21 d of preliminary phase and 14 d of feeding endophyte-infected fescue seed (E+) or endophyte-free fescue seed (E−). During the preliminary phase soybean hulls were added to the supplement instead of fescue seed. During the treatment phase of Experiment 1 steers were fed 2.15 kg supplement DM and 0.62 kg of fescue seed DM daily (Table 1), and in Experiment 2 steers were fed 2.62 kg supplement and 0.69 kg fescue seed DM daily (Table 1), with the total weight of supplement plus fescue divided equally in the AM and PM feedings. In Experiment 1, E+ supplied 2.72 mg ergot alkaloids including 1.60 mg ergovaline per steer daily and in Experiment 2, E+ supplied 3.06 mg ergot alkaloids including 2.00 mg ergovaline daily. The dose chosen was similar to that of Aldrich et al. (1993) who fed diets containing 285 μg/kg of ergovaline from fescue seed. The goal was to produce physiological changes but maintain similar intake during E− and E+ feeding to avoid confounding effects due to intake changes.

For Experiment 1 the ration was formulated to meet National Research Council (1996) nutrient requirements for CP, TDN, Ca, and P for a steer weighing 272 kg and gaining 0.6 kg/d and for Experiment 2 the ration was formulated to meet those requirements for a steer weighing 318 kg and gaining 0.6 kg/d. The fescue seed passed through a 1.1 cm screen in a hammer mill (Meadow Mills, North Wilksboro, NC) before feeding to partially disrupt the seed coat. All of the fescue seed was fed in the morning, so the amount of AM supplement fed was reduced accordingly. Steers were assigned at random to receive E− (Southern States Cooperative, Inc., Cloverdale, VA) or E+ (EverGreen Seed, LLC, Fuquay-Varina, NC) seed in a single reversal design, 4 steers fed each type of seed in period 1. The treatments were reversed in period 2. The amount of fescue seed fed was gradually increased and the amount of soybean hulls was gradually decreased during the first 3 d when seed was fed in each period (days 22–24), so d 25 was the first day on full treatment.

After 3 weeks' adaptation to facilities and protocol, steers were fed their assigned diets for 14 d then all steers returned to the adaptation diet. After 21 d, the 14-d treatment period was repeated. As in the first period, the amount of fescue seed fed was gradually increased during the first 3 d when seed was fed, so d 25 was the first day on full treatment. Steers' hair was clipped during adaptation, 5 to 7 d before the first day of feeding fescue seed with electric animal clippers that left 3 mm hair (Aesculap® Econom II, Suhl, Germany). Daily minimum, maximum, and 1200 h barn temperature and relative humidity were measured using a calibrated humidity/thermometer (Fisher Scientific, Pittsburgh, PA). These variables were measured at 3 locations in the barn and an average value was calculated. Additionally, temperature and humidity at sampling times were recorded.

**Figure 1:**
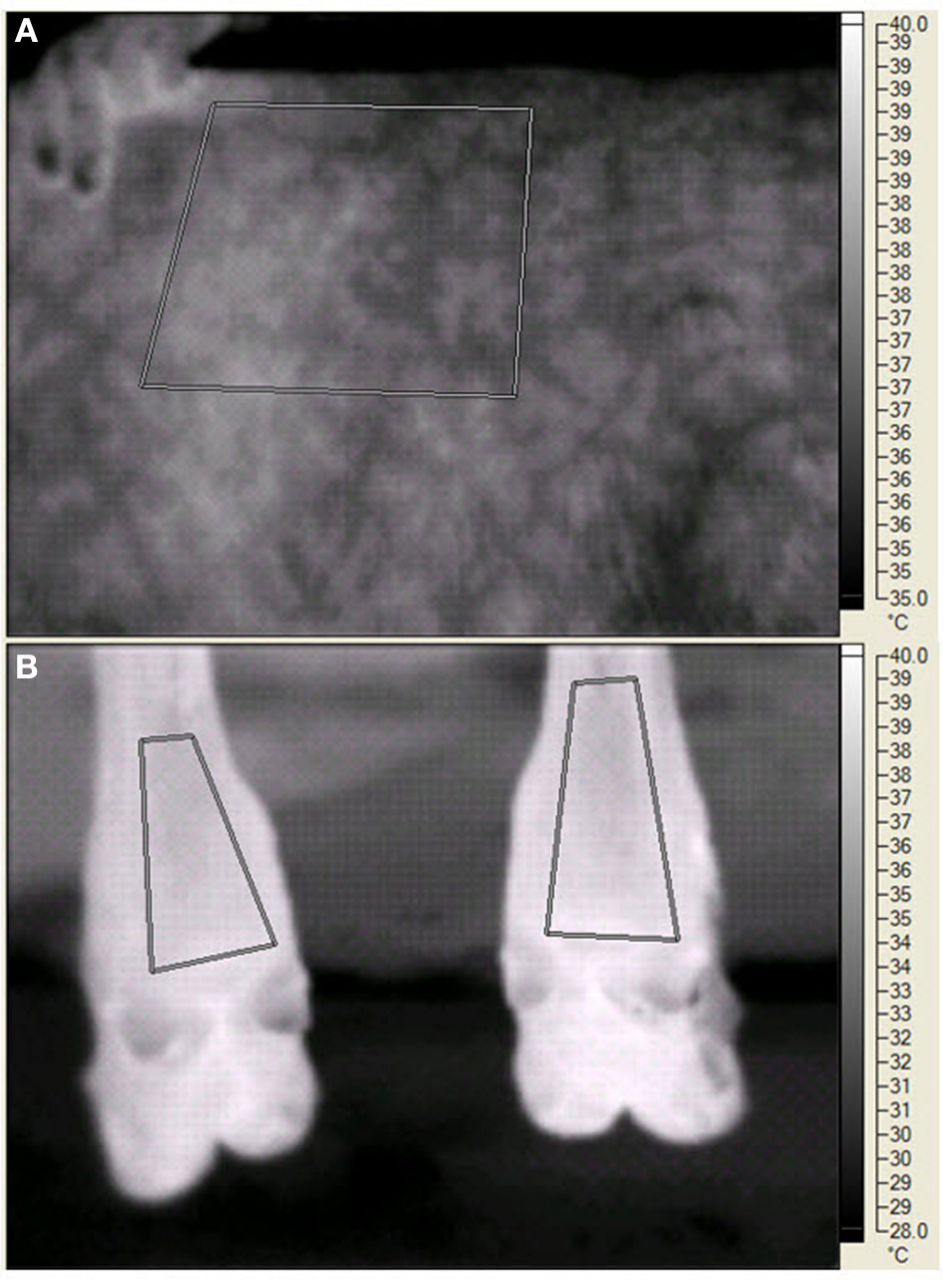
**Digital infrared images of the left side (A) and the plantar surface in the area of the large metatarsal bone in the rear legs (B) of a steer during the experiment**. The human fingers **(A)** indicate the edge of the scapula. The paralumbar fossa is to the right of the measured area. For both panels, the geometric shapes indicate areas in which temperature data were recorded. The vertical bar on the far right side of each panel indicates the temperature range (°C) and the associated light (warmer) to dark (cooler) gradient within the image.

On d 20, 25, 29, and 35 of each period palmar surface temperature (ST) in the area of the large metacarpal bone of the front legs, plantar ST in the area of the large metatarsal bone of the rear legs, and left side ST were measured by digital infrared thermal imaging. The legs were chosen because of accessibility, cleanliness, and potential relationship to ST changes in response to peripheral constriction. The left side was chosen because of the layout of the animal handling facilities and the ability to consistently measure the same area (Huntington et al., 2012). The digital thermal images were recorded with a Ti45FT (Fluke Corporation, Everett WA) with a 20 mm lens, manual focusing, 30 Hz 160 × 120 pixel focal array, and a vanadium oxide uncooled microbolometer. Emissivity was set at 0.95 and background temperature was set at 20°C. The camera has an accuracy of 2°C in the physiological temperature range, and a sensitivity of <0.1°C. Images were stored in files containing approximately 2.5 Mbyte of information that included date, time, image number, emissivity, background temperature, and visual and infrared images. Software provided by the manufacturer allowed detailed isolation of portions of the image (see Figure 1) and presented minimal, maximal, average, and the standard deviation of the pixels in the selected portion. BP and HR were measured with a 16 to 24 cm BP cuff around the tail head connected to a digital monitor (Lifesource® A&D Engineering, Inc., San Jose, CA), RT was measured with a digital thermometer (Becton, Dickenson and Co., Franklin Lakes, NJ), and RR by rib cage movement was measured for 10 s at 0730 (before collection of orts), 1230, and 1500 (before PM feeding). Data were averaged for front legs and averaged for rear legs before statistical analysis. The steers were restrained in a squeeze chute while blood was removed by jugular venipuncture, starting at 1300 h on sampling days.

In each experiment, 2 groups of 4 steers (*n* = 8 total) were staggered by 1 week to allow collection of physiological data within 30 min on each sampling day. Steers were adapted to the procedures by several practice sessions during the preliminary phases of the experiments. Two people were in the barn to collect BP and HR measures, one steer at a time in their stall, steer in standing position. One minute elapsed between deflation of the cuff and the subsequent measure. The instrumental criteria and personal experience were used to assess validity of each BP and HR measure. The steers' demeanor on a given day and time affected the number of BP and HR measures used in statistical evaluation of treatments. Of the 384 measurement episodes within steer, period, day, and time, 6 contained 2 measures, 264 contained 3 measures, 108 contained 4 measures, and 6 contained 5 measures.

In Experiment 1, a fecal grab sample was collected between 1100 and 1300 h for 3 d at the end of each fescue period for analysis of alkanes to calculate DM digestibility.

### Sample analysis

Feed samples were analyzed for nutrient content by a commercial laboratory (North Carolina Department of Agriculture, Raleigh, NC). Concentration of total alkaloids in the fescue seed was analyzed by a commercial laboratory (Agrinostics Ltd. Co., Watkinsville, GA) using an ELISA (Hill and Agee, 1994). Ergovaline concentration in the fescue seed was analyzed by a commercial laboratory (University of Missouri Veterinary Medical Diagnostic Laboratory, Colombia, MO) using HPLC (Rottinghaus et al., 1991, 1993). Serum prolactin (Bernard et al., 1993) and serum insulin (Cartiff et al., 2013) were determined by radioimmunoassay. For prolactin, the intra-assay CV was 6.8% and the inter-assay CV was 7.1%. For insulin, the intra-assay CV was 5.5% and the inter-assay CV was 8.4%. Plasma glucose was analyzed using a glucose oxidase method (Yellow Springs Instruments, Yellow Springs, OH). Concentration of hentriacontane in feed and fecal samples and calculation of DM digestibility in Experiment 1 were determined as described by Chavez et al. (2011).

### Statistical analysis of data

The Mixed procedure of SAS (SAS institute, Cary NC) was used for statistical analysis of data. The model had main effects of treatment, day, time of day, group, period, and all possible interactions of treatment, time and day. Steer, steer × period, steer × treatment, and steer × time were random effects. Except for measures in Experiment 1 of serum insulin, serum prolactin, and plasma glucose, mean values within steers across times collected on day 20 of each period were used as covariates within periods. The effect of period on baseline values was tested using a model with period, treatment, and period × treatment as the main effect. Data for intake, DM digestibility, plasma glucose, and blood hormones did not have day or time of day in the model.

## Results

**Figure 2:**
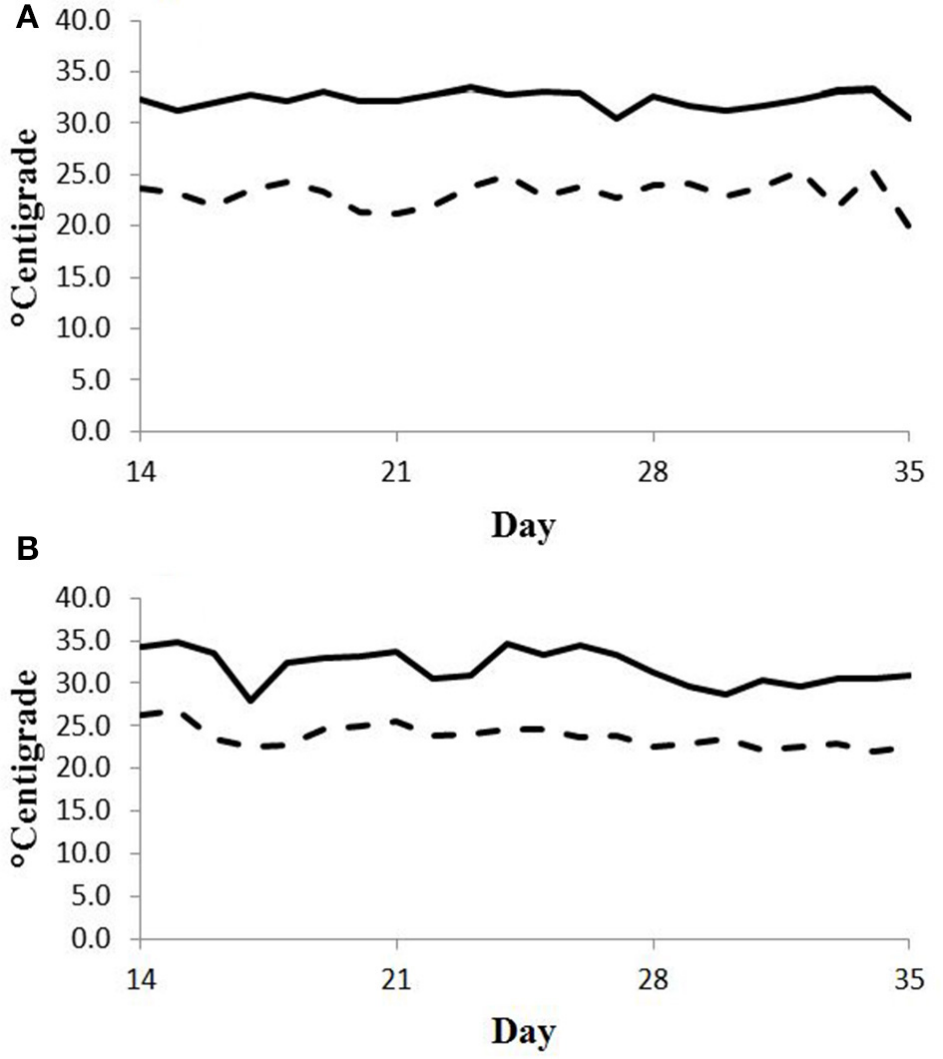
**Daily minimum (dashed line) and maximum (solid line) temperature (°C) during days 14 to 35 of period 1 (A) and period 2 (B) in Experiment 1**. Day 21 was the first day of seed feeding in each period.

**Figure 3:**
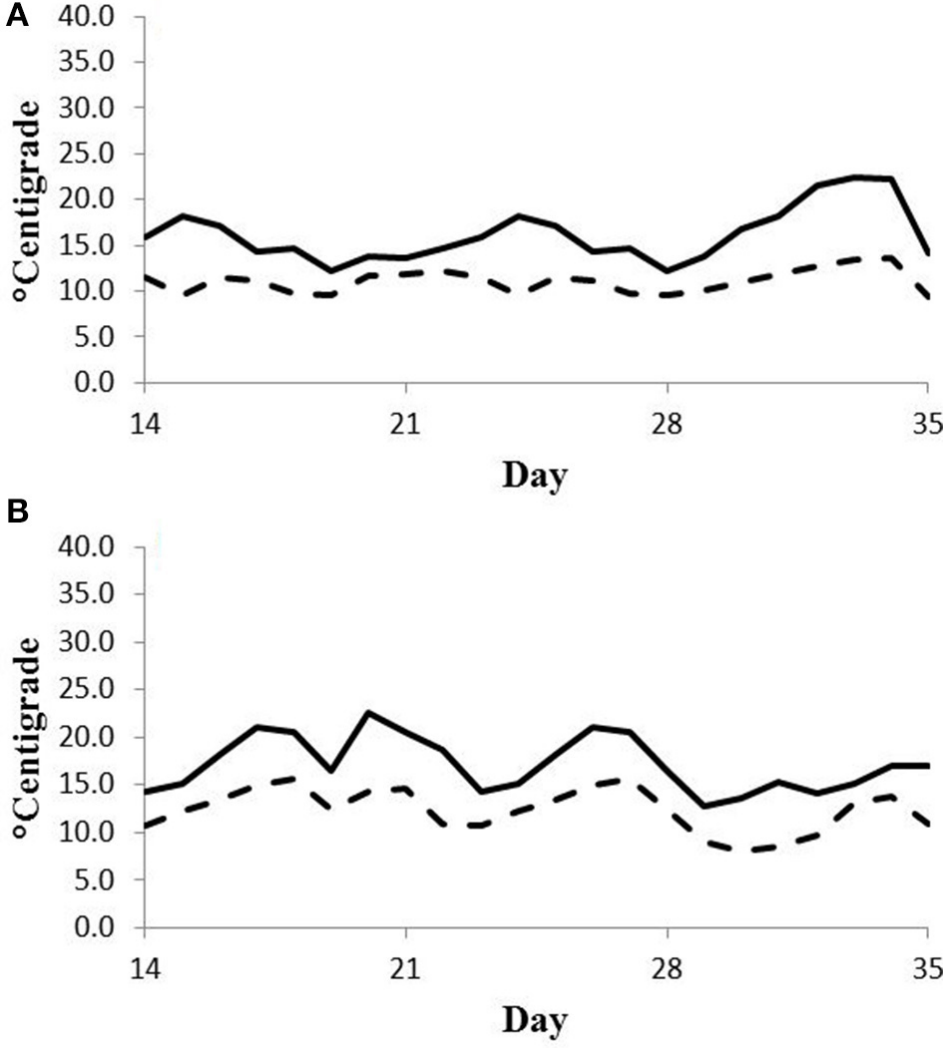
**Daily minimum (dashed line) and maximum (solid line) temperature (°C) during days 14–35 of period 1 (A) and period 2 (B) in Experiment 2**. Day 21 was the first day of seed feeding in each period.

Feed intake was not affected by treatment in either experiment (Table 2) and therefore treatment responses for E+ compared to E− are independent of intake effects. Steers were fed at a slightly restricted intake in both studies to minimize orts and remove confounding effects of intake from the responses. Steers in Experiment 1 had greater orts as a proportion of hay offered and ate slightly less DM as a proportion of BW than steers in Experiment 2. Dry matter digestibility did not differ (*P* = 0.76) for E− and E+ and was 0.584 and 0.590 g/g DM (*SE* 0.014), respectively. Barn temperature, humidity, and calculated temperature-humidity index (THI, Mader et al., 2002) indicate that steers in Experiment 1 were near or above their thermoneutral zone, and steers in Experiment 2 were within their thermoneutral zone during the experiments (Table 3). Daily minimums and maximums were within or close to those recorded during sampling times in Experiment 1 (Figure 2) and daily minimums were close to those recorded during sampling times in Experiment 2 (Figure 3). Maximum values were after sampling times in Experiment 2.

**Table 2:** **Body weight (BW), dry matter intake (DMI), and orts for steers fed endophyte-free (E−) or endophyte-infected (E+) fescue seed above (Experiment 1) or within (Experiment 2) their thermoneutral zone**.

**Item**	**Experiment 1**				**Experiment 2**			
	***E***−	***E***+	***SE***^a^	***P*** =	***E***−	***E***+	***SE***^a^	***P*** =
BW, kg	285	288	4	0.24	348	347	10	0.30
**DMI, KG/D**								
Hay	2.72	2.63	0.1	0.25	3.67	3.68	0.06	0.82
Total	5.44	5.33	0.1	0.18	6.98	6.99	0.06	0.82
Total g/kg BW	19.1	18.5	0.4	0.12	20.2	20.3	0.5	0.40
Orts g/kg hay	174	197	27.5	0.28	60	57	14.2	0.82

a n = 8.

**Table 3:** **Barn temperature, relative humidity, and temperature-humidity index (THI) for steers fed endophyte-free (E−) or endophyte-infected (E+) fescue seed above (Experiment 1) or within (Experiment 2) their thermoneutral zone**.

**Item**	**Experiment 1**				**Experiment 2**			
	**Period 1**		**Period 2**		**Period 1**		**Period 2**	
	**Mean**	**STD**	**Mean**	**STD**	**Mean**	**STD**	**Mean**	**STD**
**TEMPERATURE, °C**								
Minimum^a^	23.2	1.2	24.0	1.2	11.9	1.9	11.9	2.3
Maximum^a^	32.4	0.8	32.2	1.9	17.4	4.1	17.4	2.8
1200^a^	30.3	1.3	30.0	2.7	15.8	4.0	15.6	2.8
Sampling days^b^								
0730	24.5	1.3	24.3	0.8	12.2	2.1	12.5	1.9
1230	30.9	1.1	29.1	2.8	15.5	3.6	15.4	3.2
1530	30.6	2.0	30.1	1.7	16.2	4.0	16.4	3.5
**RELATIVE HUMIDITY**, %								
Minimum^a^	46.5	10.7	50.5	10.9	46.6	10.7	53.3	13.3
Maximum^a^	81.3	6.7	86.2	6.9	77.9	7.3	83.1	10.6
1200^a^	58.3	10.2	64.1	14.7	57.3	13.2	64.6	13.9
Sampling days^b^								
0730	74.6	7.7	79.8	8.6	62.9	7.2	64.5	13.7
1230	57.4	10.1	65.1	16.0	54.3	8.8	58.8	11.3
1530	56.5	15.2	57.6	14.5	46.7	7.7	54.2	11.8
**THI^c^**								
Minimum^a^	69.0	2.0	70.3	1.7	54.7	2.5	54.8	2.9
Maximum^a^	86.9	1.6	87.4	2.7	62.7	6.6	63.0	4.7
1200^a^	79.9	2.2	80.1	2.5	59.7	5.5	59.8	4.2
Sampling days^b^								
0730	73.5	2.4	73.7	1.6	54.7	3.0	55.2	2.7
1230	80.2	2.6	78.9	3.1	59.3	4.8	59.3	4.7
1530	79.5	1.9	79.3	2.0	60.0	4.9	60.8	5.0

a Data from the last 16 d of each period.

b d 20, 25, 29, and 35 of each period.

c THI = 0.8Ta + [(0.01 RH × (Ta−14.3)] + 46.3 where Ta = ambient temperature, °C (Mader et al., 2002).

### Baseline data

In both experiments, there were few differences (*P* < 0.21) between baseline values collected on d 20 of each period that were used as covariates in the statistical model. In Experiment 1 there were differences (*P* < 0.05) for left side ST standard deviation (0.38 vs. 0.31°C) and trends (0.05 < *P* < 0.07) for systolic pressure (117 vs. 103 mm Hg) and diastolic pressure (52 vs. 47 mm Hg). In Experiment 2 there were differences (*P* < 0.05) for RR (31 vs. 24 breaths/min) and RT (38.2 vs. 38.5°C). In no case was there a period × treatment interaction supporting the fact that there was no carryover between periods for the variables measured.

### Responses to days of feeding fescue seed

In Experiment 1 there was a trend (*P* < 0.10) for increased left side ST, but in Experiment 2 STs decreased (*P* < 0.05) with days of feeding fescue seed (Table 4). There were trends (*P* < 0.10) for day × treatment interactions; diastolic BP increased in Experiment 1 for E+ but did not change for E− with days of feeding fescue seed, and there was a decrease in systolic BP in Experiment 2 for E− whereas systolic BP increased for E+ with days of feeding fescue seed. The difference between systolic and diastolic BP decreased for E− but increased for E+ with days of feeding fescue seed in Experiment 2 (Table 4). The day × treatment interaction (*P* < 0.05) for RR in Experiment 1 was caused by a greater increase for E+ than E− with days of feeding fescue seed (Table 4). The trend (*P* < 0.10) for the same interaction in Experiment 2 (Table 4) was caused by a lesser decrease in RR for E+ than E− with days of feeding fescue seed. The ST of rear legs tended (*P* < 0.10) to fluctuate more for E+ than E− with days of feeding fescue seed in Experiment 2, but there was no interaction in Experiment 1 (Table 4).

**Table 4:** **Blood pressure, heart rate (HR), respiration rate (RR), rectal temperature (RT), and surface temperature for steers fed endophyte-free (E−) or endophyte-infected (E+) fescue seed for 14 d above (Experiment 1) or within (Experiment 2) their thermoneutral zone**.

**Item**	**Day of feeding fescue seed^a^**								
	**4**		**8**		**14**			***P* =**	
	***E*−**	***E*+**	***E*−**	***E*+**	***E*−**	***E*+**	***SE***	**Day**	**Day × Trt^b^**
**EXPERIMENT 1**									
Systolic, mm Hg	104.2	97.7	104.1	102.0	104.8	101.6	2.9	0.63	0.49
Diastolic, mm Hg	48.7	48.0	48.3	54.1	47.5	52.4	1.9	0.26	0.08
S–D^b^, mm Hg	54.5	56.0	54.8	49.0	56.2	50.2	2.5	0.81	0.78
HR, beats/min	62	57	62	57	63	58	1	0.17	0.95
RR, breaths/min	31	30	32	34	32	50	1.8	0.01	0.01
RT, °C	38.3	38.3	38.3	38.5	38.3	38.9	0.1	0.01	0.01
Surface Temperature, °C									
Rear legs^c^	33.8	33.9	33.5	34.3	34.1	34.4	0.24	0.11	0.32
Front legs^c^	34.8	35.1	34.9	35.0	35.1	35.4	0.21	0.35	0.82
Front–rear legs	1.07	1.18	1.41	0.73	0.91	0.93	0.21	0.54	0.07
Left side	36.4	36.8	36.6	37.0	36.9	37.4	0.2	0.08	0.99
**EXPERIMENT 2**									
Systolic, mm Hg	116.1	103.7	114.9	108.3	110.0	106.7	3.2	0.45	0.07
Diastolic, mm Hg	57.1	58.9	56.4	59.7	55.7	56.6	1.9	0.49	0.72
S-D, mm Hg	56.7	47.2	56.1	50.9	52.0	52.4	2.6	0.79	0.02
HR, beats/min	74	66	72	65	72	65	1.5	0.32	0.91
RR, breaths/min	27	26	22	23	22	23	0.6	0.01	0.09
RT, °C	38.3	38.1	38.2	38.1	38.2	38.1	0.1	0.74	0.54
Surface Temperature, °C									
Rear legs^c^	25.1	25.8	23.4	22.6	23.2	23.8	0.48	0.01	0.10
Front legs^c^	28.6	28.7	26.6	26.7	26.6	26.8	0.36	0.01	0.97
Front–rear legs	3.64	2.83	3.30	4.05	3.74	2.94	0.34	0.27	0.01
Left side	31.9	32.4	30.4	31.5	30.6	30.7	0.35	0.01	0.18

a Periods lasted 35 days; fescue seed was fed for 14 days (d22–d35) of each period; n = 8 per treatment.

b Trt, treatment; S–D, systolic–diastolic blood pressure.

c Surface temperature of rear legs represents the plantar surface in the area of the large metatarsal bone; surface temperature of front legs represents the palmar surface in the area of the large metacarpal bone.

The day × treatment interaction of ST difference between front and rear legs (*P* < 0.01) in Experiment 2 reflected similar decreased surface ST of front legs for E− and E+ and of rear legs for E− while ST of rear legs for E+ showed greater fluctuation with days of feeding fescue seed (Table 4).

Serum insulin concentration was greater (*P* < 0.05, Experiment 1) or tended (*P* = 0.07, Experiment 2) to be greater for E+ than E− and plasma glucose concentration was greater (*P* < 0.01) for E+ than E− in Experiment 2 (Table 5). Serum prolactin concentrations were numerically lower for E+ than E− in both experiments, but variation among steers precluded statistical significance. The average prolactin concentration for E+ was 26% of E− in Experiment 1 and 60% of E− in Experiment 2.

**Table 5:** **Serum insulin, serum prolactin, and plasma glucose concentrations for steers fed endophyte-free (E−) or endophyte-infected (E+) fescue seed above (Experiment 1) or within (Experiment 2) their thermoneutral zone**.

**Item**	**Day of feeding fescue seed^a^**									
	**4**		**8**		**14**			***P* =**		
	***E*−**	***E*+**	***E*−**	***E*+**	***E*−**	***E*+**	***SE***	**Trt^b^**	**Day**	**Day × Trt**
**EXPERIMENT 1**										
Prolactin, ng/mL	175	75	160	24	117	21	86	0.37	0.03	0.44
Insulin, uIU/mL	14.1	18.4	16.5	20.9	17.6	23.3	1.7	0.02	0.03	0.88
Glucose, mM	3.53	3.71	3.58	3.70	3.63	3.74	0.02	0.31	0.40	0.76
t**EXPERIMENT 2^c^**										
Prolactin, ng/mL	92	55	54	29	67	44	13.1	0.14	0.30	0.84
Insulin, uIU/mL	19.1	20.5	20.1	20.5	20.6	23.1	1.2	0.07	0.76	0.18
Glucose, mM	4.01	4.28	4.12	4.25	4.07	4.33	0.05	0.01	0.45	0.19

a Periods lasted 35 days; fescue seed was fed for 14 days (d22–d35) of each period.

b Trt, treatment.

c Day 20 values were used as a covariate in Experiment 2.

### Time of day and treatment responses

Systolic BP decreased (*P* < 0.05, Experiment 1) with time of day (Table 6) and tended to decrease (*P* < 0.10, Experiment 2) with E+. Diastolic BP decreased (*P* < 0.05) with time of day in Experiment 2, and was greater for E+ than E− in Experiment 1. In both experiments, systolic – diastolic BP difference was lesser for E+ than E−, with a trend for greater decrease with time of day for E+ than E− in Experiment 1 (time of day × treatment interaction, *P* < 0.10). HR increased with time of day and was lesser for E+ than E− in both experiments (Table 6). RR increased (*P* < 0.01) with time of day in both experiments. It was greater (*P* < 0.01) for E+ than for E− in Experiment 1 with a greater increase for E+ than E− with time of day in Experiment 1 (time of day × treatment interaction, *P* < 0.05). In both experiments, RT and STs of legs and the left side increased (*P* < 0.05) with time of day, but there were no time of day × treatment interactions (Table 6). In Experiment 1, RT and left side ST were greater (*P* < 0.05) for E+ than E−.

**Table 6:** **Blood pressure, heart rate (HR), respiration rate (RR), rectal temperature (RT), and surface temperature at different times of day for steers fed endophyte-free (E−) or endophyte-infected (E+) fescue seed above (Experiment 1) or within (Experiment 2) their thermoneutral zone**.

**Item**	**Time of day**									
	***E*−**			***E*+**				***P* =**		
	**0730**	**1230**	**1530**	**0730**	**1230**	**1530**	***SE***	**Trt^a^**	**Time^a^**	**T × T^a^**
**EXPERIMENT 1**										
Systolic, mm Hg	106.9	106.1	100.0	105.0	97.9	98.5	2.8	0.18	0.03	0.17
Diastolic, mm Hg	49.7	48.1	46.7	51.4	51.3	51.7	1.9	0.03	0.79	0.57
S-D^a^, mm Hg	56.2	57.0	52.3	54.5	47.5	47.7	2.41	0.05	0.03	0.08
HR, beats/min	60.2	63.8	63.1	55.1	58.1	58.4	0.96	0.01	0.01	0.84
RR, breaths/min	27	33	35	29	41	44	2	0.01	0.01	0.03
RT, °C	38.1	38.3	38.5	38.2	38.6	38.9	0.1	0.05	0.01	0.16
Surface Temperature, °C										
Rear legs^b^	31.8	34.9	34.7	31.9	35.2	35.4	0.24	0.13	0.01	0.32
Front legs^b^	33.5	35.8	33.5	33.7	35.6	36.0	0.21	0.29	0.01	0.25
Front–rear legs	1.67	0.84	0.87	1.80	0.42	0.63	0.21	0.41	0.01	0.32
Left side	35.2	37.0	37.5	35.8	37.3	38.1	0.19	0.05	0.01	0.83
**EXPERIMENT 2**										
Systolic, mm Hg	114.2	111.4	110.5	108.2	106.2	104.1	3.1	0.07	0.22	0.86
Diastolic, mm Hg	58.5	55.7	55.0	60.7	56.1	58.4	1.8	0.32	0.04	0.61
S-D, mm Hg	53.7	56.7	54.5	49.9	52.6	48.1	2.4	0.04	0.13	0.73
HR, beats/min	69.6	74.8	73.2	63.3	66.9	66.0	1.5	0.01	0.02	0.74
RR, breaths/min	22	25	25	22	24	24	1	0.98	0.01	0.75
RT, °C	38.0	38.2	38.4	38.0	38.2	38.3	0.1	0.49	0.02	0.78
Surface Temperature, °C										
Rear legs^b^	21.6	25.0	25.1	21.5	25.3	25.3	0.48	0.76	0.01	0.96
Front legs^b^	25.7	28.0	28.1	25.6	28.2	28.4	0.35	0.71	0.01	0.81
Front–rear legs	4.2	3.5	3.0	4.0	2.8	3.0	0.35	0.28	0.01	0.52
Left side	29.6	31.3	32.0	30.0	32.1	32.4	0.35	0.18	0.01	0.58

a Trt, treatment; Time, time of day; T × T, treatment × time of day; S–D, systolic–diastolic blood pressure.

b Surface temperature of rear legs represents the plantar surface in the area of the large metatarsal bone; surface temperature of front legs represents the palmar surface in the area of the large metacarpal bone.

## Discussion

Scharf et al. (2011) reported the critical point for ambient temperature to increase core body temperature of growing cattle in feedlots at about 25°C. Hahn (1999) suggested a thermal stress threshold of 25°C for growing cattle fed *ad libitum* which coincided with decreased feed intake and 21°C as the threshold for increased RR. Scharf et al. (2011) observed also that cattle showed nighttime recovery to decrease core body temperature. In both of the current experiments RT, RR, and STs were at their minimum at the 0730 sampling for both E− and E+. This sampling time had ambient temperature and THI that were close to the minimum values. RT, RR, and ST cycled in all steers and an increase in RR, RT, and left side ST due to E+ was observed only when steers were housed above their thermoneutral zone. The increases due to E+ were associated with THI of around 74–80, above 75 which is the point suggested for using thermal stress-limiting measures (Hahn, 1999).

Routes of heat flow from the animal to the environment are conduction, convection, and radiation which depend on thermal gradients within the animal and between the animal and the environment; and evaporation which depends on humidity. Skin temperature below 35°C provided a large enough temperature gradient between the body core and the skin to use all 4 routes of heat exchange (Collier et al., 2006). Mechanisms for heat dissipation in response to thermal stress include increased RR, increased peripheral vasodilation, increased skin temperature, and increased sweat rate. Blood flow to the periphery increases to increase heat loss via conduction and convection. Hair coat can reduce heat flow via these two routes. Heat stress increases sweating rate and RR. Evaporation is the major route of heat loss as ambient temperature approaches skin temperature (Hansen, 2004; Scharf et al., 2010). In Experiment 1, steers were housed above their thermoneutral zone, and left side ST was above 35°C which may have reduced the effectiveness of heat transfer from the body core to the skin and resulted in increased RR.

Scharf et al. (2010) observed increased RR, RT, skin temperature, and sweat rate in Angus or Romosinuano steers housed at temperatures above the thermoneutral zone, cycling from 26°C during the night to 36°C during the day, compared to thermoneutral housing (21°C). Skin temperatures were highly correlated with ambient temperatures. Decreased sweat rate was correlated with increased RT during heat stress for Angus cattle (Scharf et al., 2010). STs in both of the current experiments were consistent with a correlation between skin temperature and ambient temperature.

Steers receiving E+ had greater left side ST than those receiving E− when housed above the thermoneutral zone suggesting vasodilation and greater transfer of core body heat to the periphery which should increase heat loss by conduction and convection. The maximum ambient temperature was 32.3°C. Previous studies in which heat stress was constant showed no change in ST due to short-term feeding of E+ (Rhodes et al., 1991; Al-Haidary et al., 2001; Koontz et al., 2012) or decreased ST due to a single injection of ergot alkaloids (Browning and Leite-Browning, 1997; Browning, 2000). Changes in ST reflect changes in ambient temperatures, hair coat, and peripheral blood flow. We clipped hair in an effort to minimize hair coat effect among steers or in response to treatment. The thermal imaging camera provides data on minimum, maximum, average and standard deviation of ST. Previous work with the same camera showed an inverse correlation between average and the standard deviation of side ST in bulls not exposed to toxic fescue (Huntington et al., 2012), indicating that thermal imaging may detect variation in ST due to thermal patterns created by vasodilation. In the current experiments correlations between mean and standard deviations of ST within experiments (data not shown) were not statistically significant (*P* < 0.10). Steer's hair in our study was not clipped as close to the skin as it was for the Angus bulls in Huntington et al. (2012). ST of front legs was consistently greater than ST of rear legs although the difference in temperature between them declined with time of day. Lack of interactions between days of feeding fescue or time of day with treatment indicates that either front or rear legs could be used to evaluate changes in ST.

RR increased to a greater rate in response to E+ in conditions above the thermoneutral zone in Experiment 1. It is possible that other avenues of heat dissipation were not responding to environmental conditions. Increased RR, sweat rate, and peripheral vasodilation contribute to internal body temperature response to heat stress (Scharf et al., 2010). Decreased skin vaporization was observed in steers housed at 32°C and fed a similar dose of E+ to that fed in the present study compared to steers fed E− (Aldrich et al., 1993). RT was greater for E+ than E− in the current study.

HR decreased due to E+ in cattle housed at both ambient temperatures. Decreased HR should result in decreased BP if other variables that affect pressure are unchanged (Melbin and Detweiler, 1993). Systolic-diastolic pressure difference, pulse pressure, decreased for steers eating E+ in both environments but for different reasons. Under thermoneutral conditions in Experiment 2, there was a trend for decreased systolic BP whereas under conditions above the steers' thermoneutral zone in Experiment 1 diastolic BP increased. Increased diastolic pressure may reflect increased peripheral resistance (Ganong, 1975) due to the known effects of ergot alkaloids from fescue to promote vasoconstriction in some vascular tissues (Oliver et al., 1998; Aiken et al., 2007, 2009; Klotz et al., 2007). These effects may be more pronounced due to regulatory changes in response to thermal stress which promote increased blood flow to the skin than under conditions of basal skin blood flow. At greater doses of alkaloids than that used in the current experiments, vasodilation in response to thermal stress may be more limited due to alkaloid-induced vasoconstriction.

Effects of ergot alkaloids in the fescue seed on decreasing HR, increasing diastolic BP, and RR are consistent with other reports in cattle (Browning and Leite-Browning, 1997; Browning, 2000; Koontz et al., 2012) and sheep (McLeay et al., 2002). However, Rhodes et al. (1991) found no effects of consuming 1.14 mg ergovaline/d on HR, BP or skin temperature of small (88 kg BW) Holstein steers housed at 32°C and restricted to intake equal to 25 g/kg BW. Aiken et al. (2007) observed decreased systolic BP, diastolic BP, and HR in response to ergot alkaloids in beef heifers (375 kg BW), housed below their upper critical temperature and consuming 7.65 mg ergovaline/d. Intake of the heifers tended (*P* < 0.15) to be lesser for those consuming alkaloids (9 kg DM/d) than control heifers (10.7 kg/d). Baseline values for the heifers (measured with a pressure cuff on the tail head) for systolic BP (143 mm Hg), diastolic BP (77 to 86 mm Hg), and HR (106 beats/min) were greater than those measures in steers in the current experiments before (data not shown) or during feeding E− or E+. Aiken et al. (2007) did not describe details of location of heifers during measures, or adaptation to BP procedures. The use of a pressure cuff on the tail head (Browning and Leite-Browning, 1997) in animals accustomed to its use and measured in their usual pens provides credible values for HR and BP similar to those reported for cattle (Rhodes et al., 1991) and sheep (McLeay et al., 2002) with indwelling pressure monitors. Koontz et al. (2012) observed a trend toward short-term increases in diastolic BP in response to toxic fescue seed extract administered to the rumen. The steers in Koontz et al. (2012) were evaluated within and above their upper critical temperature, but feed intake decreased in response to intraruminal dosing of extract from E+ and increased ambient temperature from 22 to 32°C. Our results demonstrate cardiovascular effects from E+ independent of potential effects of DMI at ambient temperatures within or above the animals' thermoneutral zone. Increased serum insulin concentrations in both experiments, and either no change (Experiment 1) or increased plasma glucose concentrations (Experiment 2) in response to E+ indicate changes in homeorhetic control of glucose metabolism which could be linked to insulin resistance and subsequent effects on glucose metabolism when the steers were fed E+.

Ergot alkaloids from fescue seed affect the cardiovascular system of steers separately from effects of feed intake or environmental temperature. Ergot alkaloids interact with ambient temperatures above the steers' thermoneutral zone to exacerbate the symptoms of hyperthermic stress. Based on the data from these studies, there may be insulin-dependent glucose metabolism changes in response to ergot alkaloids.

### Conflict of interest statement

The authors declare that the research was conducted in the absence of any commercial or financial relationships that could be construed as a potential conflict of interest.
